# Listeriosis Infection in Pregnancy: A Case Series

**DOI:** 10.7759/cureus.74135

**Published:** 2024-11-21

**Authors:** Salwa Ahmed, Fatima Ba Khamis, Komal Hazari, Deemah K Harb, Widad Abdelkareem, Laila Alhubaishi

**Affiliations:** 1 Obstetrics and Gynecology, Dubai Health, Dubai, ARE; 2 Internal Medicine, Latifa Women and Children's Hospital, Dubai, ARE; 3 Obstetrics and Gynecology, Latifa Women and Children's Hospital, Dubai, ARE

**Keywords:** fetal listeria infection, listeria infection, listeria infection in pregnancy, listeriosis, listeriosis in pregnancy

## Abstract

Listeriosis is a rare foodborne febrile illness that has a unique predilection for the pregnant population. Listeriosis during pregnancy can cross the placenta, resulting in vertical transmission to the fetus which frequently results in adverse pregnancy outcomes. This is a case series on three pregnant patients who were treated for listeriosis at Latifa Women and Children’s Hospital. All three patients reported in this series had no medical comorbidities or pregnancy risk factors; they presented with fever and abdominal and/or back pain; and of significance, none of the patients reported a history of gastrointestinal tract symptoms. Two cases were in the second trimester and one in the third trimester. All cases resulted in fetal loss with 100% fetal mortality. There was no maternal mortality or long-term morbidity in any of the patients.

## Introduction

Listeriosis is a rare and severe foodborne infection caused by the microorganism *Listeria monocytogenes*, which is a Gram-positive facultative intracellular pathogen [1]. Pregnant women are approximately 18 times more likely to contract listeriosis than the general population [2]. According to the World Health Organization (WHO), listeriosis during pregnancy accounted for nearly 43% of the total listeriosis cases [3]. Pregnant women may be able to reduce the risk of *Listeria *infection by following dietary guidelines recommended by the Centers for Disease Control and Prevention (CDC). Clinical features are nonspecific and include fever, abdominal or back pain, diarrhea, nausea and/or vomiting, and myalgia [4]. Maternal infection may be mild and resolve without therapy [5] or may result in severe infection with bacteremia and meningitis; however, central nervous system (CNS) involvement in pregnancy is relatively uncommon [6]. Outcomes for pregnant women with treated *Listeria* infection are typically good; however, fetal and neonatal infections can be severe, leading to fetal loss, preterm labor, neonatal sepsis, meningitis, and death [7]. Antenatal maternal treatment is associated with less severe listeriosis in the neonate [8].

*Listeria monocytogenes *crosses the placental barrier, resulting in fetal infection, which may result in granulomatosis infantiseptica. Infants with this condition have disseminated abscesses and/or granulomas in multiple organs; most neonates with this condition are stillborn or die soon after birth [9]. Placental histopathology is distinctive; there is a pronounced chorioamnionitis and an acute villitis with abscess formation; and the organisms are frequently seen within the amnion epithelium [5]. 

A series that included more than 200 cases of pregnant patients with listeriosis found that approximately 20% of pregnancies resulted in spontaneous abortion or stillbirth, and approximately 66% of surviving infants developed neonatal listeriosis [4]. Adverse fetal and neonatal outcomes are less common when infection occurs at a later gestational age [10]. A 10-year cohort study involving more than 150 pregnant patients with listeriosis found that fetal loss was 100% when infections occurred in the first trimester, compared with 70% when infection occurred in the second trimester, and less than 5% when infection occurred in the third trimester [11]. Comparably, in another series of 11 cases of pregnancy-associated listeriosis, there were two cases in which infection occurred before the third trimester (at 17 and 18 weeks), and both resulted in fetal death, one following spontaneous abortion and the other immediately after delivery. The remaining nine cases occurred in the third trimester, and six of the patients delivered preterm and had neonates with listeriosis, whereas the remaining three full-term infants were not infected [12]. This case series describes the clinical course and pregnancy outcome of the three cases of listeriosis in pregnancy that were treated in Latifa Women and Children Hospital. 

## Case presentation

Case 1

A 26-year-old primigravida presented to the emergency department at 18 weeks and six days gestation with abdominal pain and vaginal leaking a few hours prior to presentation associated with tactile fever for the past three days. Prior to presentation, her pregnancy was uneventful. 

Upon presentation, she was febrile (Table 1). On examination, blood-stained leaking was noted, ultrasound examination was suggestive of oligohydramnios, and her labs were suggestive of infective pathology (Table 2).

**Table 1:** Summary of clinical cases PPROM: preterm premature rupture of membranes; IUFD: intrauterine fetal death

		Patient 1	Patient 2	Patient 3
Patient (age in years)		27	28	29
Week of gestation at onset		18	17	33
Week of gestation at diagnosis		2 days postpartum	4 days postpartum	1 day postpartum
Clinical presentation		Abdominal pain, per vaginal leaking, fever (37.9°C)	Lower abdominal pain, tactile fever, missed abortion	Abdominal pain, fever (38.8°C), absent fetal movements
Treatment of maternal infection	Initial treatment	Ampicillin, clindamycin, and gentamicin	Ampicillin, clindamycin, and gentamicin	Piperacillin-tazobactam and amikacin, deescalated to ampicillin and amikacin
	Discharge treatment	Trimethoprim-sulfamethoxazole	Augmentin	Augmentin
Maternal outcome		Discharged in stable condition	Discharged in stable condition	Discharged in stable condition
Obstetric outcome		PPROM, chorioamnionitis, medical-induced abortion	Missed abortion managed medically then surgically	IUFD

**Table 2:** Laboratory findings on presentation for each case ALT: alanine transaminase; AST: aspartate transferase; PT: prothrombin time; PTT: partial thromboplastin time; INR: international normalized ratio

Laboratory findings on presentation	Patient 1	Patient 2	Patient 3	Reference range
White cell count	16.7 x 10^8^/uL	16.0 x 10^3^/uL	25.4 x 10^3^/uL	5.6-16.0 x 10 ^3^/uL
Absolute neutrophil count	12.5 x 10^3^/uL	11.1 x 10^3^/uL	20.3 x 10^3^/uL	3.8-13.1 x 10^3^/uL
Absolute monocyte count	2.4 x 10^3^/uL	1.2 x 10^3^/uL	1.7 x 10^3^/uL	0.1-1.4 x 10^3^/uL
Absolute lymphocyte count	1.8 x 10^3^/uL	3.4 x 10^3^/uL	3.30 x 10^3^/uL	0.9-3.9 x 10^3^/uL
C-reactive protein	184.9 mg/L	61 mg/L	104.1 mg/L	<0.3 mg/L
Procalcitonin	0.28 ng/mL	0.11 ng/mL	0.32 ng/mL	<0.05 ng/mL
ALT	31 U/L	43 U/L	30 U/L	<40 U/L
AST	Not done	17 U/L	Not done	<40 U/L
Bilirubin total	1.3 mg/dL	0.2 mg/dL	0.8 mg/dL	<1.2 mg/dL
PT	15.4 secs	14.9 secs	14.2 secs	<14 secs
PTT	38.8 secs	43 secs	43.8 secs	<38 secs
INR	1.22	1.18	1.10	0.80-1.10

**Figure 1:**
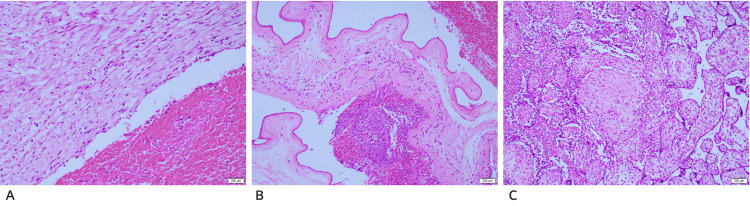
Placental histopathology slides of patient 1 A shows umbilical vessels with scattered neutrophils in the wall. B shows the membranes with scattered acute inflammatory cells. C shows the placenta with evidence of acute villitis and intervillositis

A working diagnosis of chorioamnionitis was made, and she was started on empirical triple antibiotics (ampicillin, clindamycin, and gentamicin) for three days. She aborted a 259 g fetus five hours later. She had significant bleeding, and the placenta was in situ, so she was taken for an evacuation of retained products of conception under general anesthesia, which was uneventful. Her placenta was sent for histopathology which was reported as focal acute villitis and areas of hemorrhage, and the membranes showed moderate to severe chorioamnionitis (Figure 1).

Postoperatively she remained afebrile, and triple antibiotics were deescalated to oral sulfamethoxazole/trimethoprim. Two days postpartum, she was diagnosed with *Listeria *bacteremia based on the culture report (Table 3). She was discharged home in stable condition 48 hours after admission. The patient has not followed up in our facility.

**Table 3:** Culture results for each case

Cultures results	Patient 1	Patient 2	Patient 3
Urine culture	No growth	No growth	No growth
High vaginal swab	*Listeria monocytogenes* positive	Not done	No growth
Blood culture	*Listeria monocytogenes* positive	Not done	*Listeria monocytogenes* positive

Case 2

The patient was a 26-year-old previously healthy female, P1 + 0, with a previous normal vaginal delivery. She was referred to our hospital from another facility at 19 weeks and four days gestation with a history of fever associated with lower abdominal pain of one-week duration (Table 1) and a bedside ultrasound scan showing no fetal cardiac activity.

Her labs and fever were suggestive of a bacterial infection (Table 2); hence, her working diagnosis was pyelonephritis or chorioamnionitis. The patient was started on triple antibiotics (ampicillin, clindamycin, and gentamicin). She was started on medical management of second-trimester miscarriage and aborted a 278 g fetus five hours later with an incomplete placenta. Hence, she was taken for evacuation of retained products under general anesthesia.

Postoperatively, she was continued on the same triple antibiotics for a total of 72 hours, and she remained afebrile. She was discharged three days postoperatively on oral amoxicillin 875 mg potassium clavulanate 125 mg, one tablet BD for seven days. 

**Figure 2:**
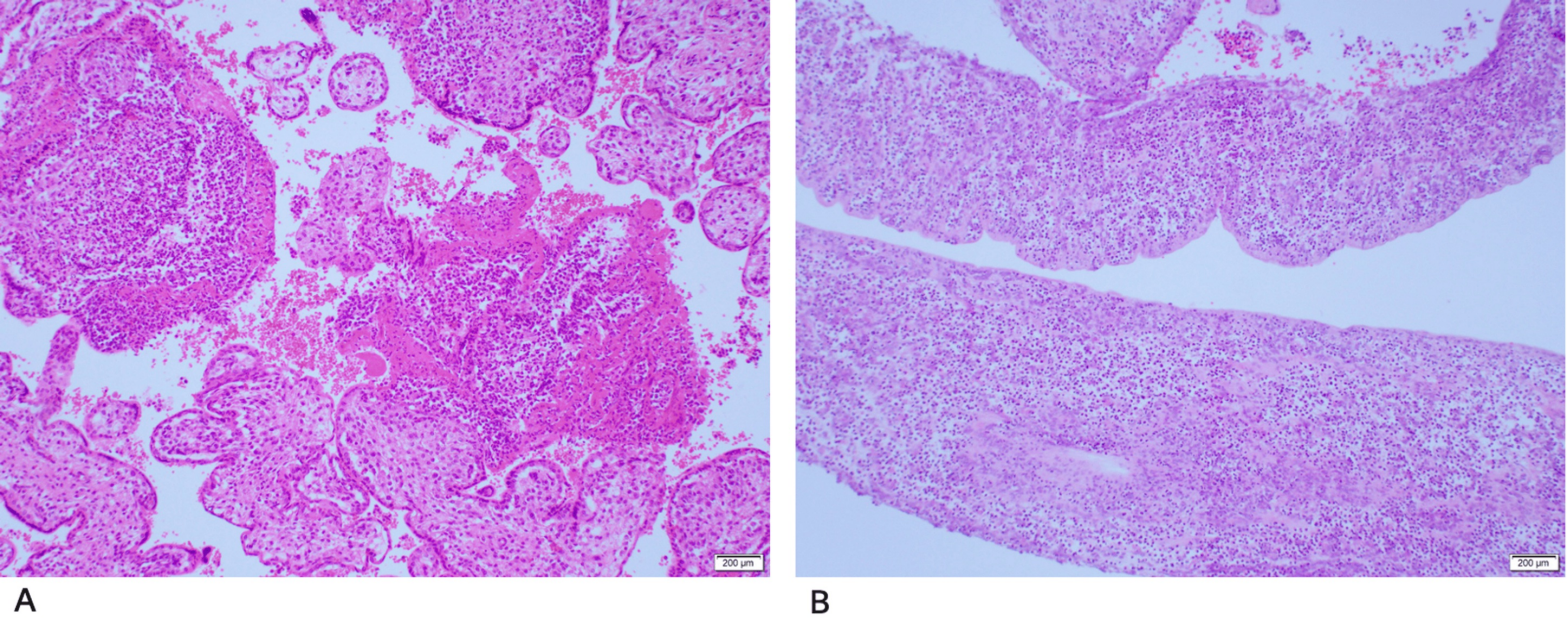
Placental histopathology slides of patient 2 A shows the placenta with acute villitis and intervillositis. B shows the membranes with extensive acute inflammatory cells

The placental histopathology showed acute villitis with microabscess formation suggestive of *Listeria *infection (Figure 2). However, high vaginal swab and blood culture were not collected for this patient to confirm the diagnosis (Table 3). Nonetheless, the diagnosis is most probably listeriosis given the clinical course and histopathology report. She was seen in routine follow-ups, and she was doing well with no complaints.

Case 3

A 29-year-old primigravida presented to the obstetric emergency at 33 weeks and one day gestation with complaints of fever and absent fetal movements of one-day duration (Table 1). An ultrasound scan confirmed a single fetus with no fetal cardiac activity. Because the patient’s septic markers were high (Table 2), she was started on piperacillin-tazobactam and amikacin. She eventually progressed spontaneously and delivered a 2480 g fetus vaginally after one day of admission. 

**Figure 3:**
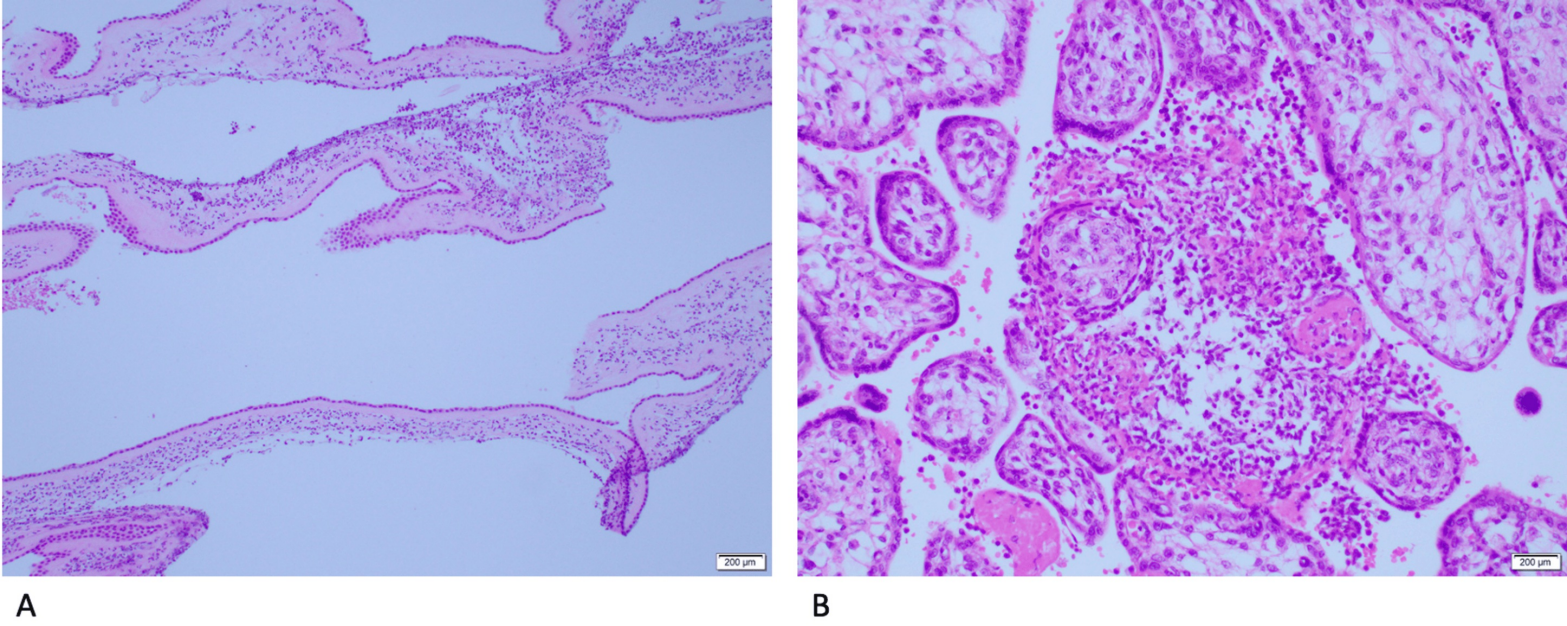
Placental histopathology slides of patient 3 A shows the membranes with extensive acute inflammatory cells. B shows the placenta with acute villitis and intervillositis

Shortly after her delivery, blood culture showed *Listeria monocytogenes* (Table 3), and Tazocin (received it for one day) was deescalated to ampicillin in which she took for three days (Table 1). She was discharged on oral Augmentin for five days. Histopathology of the placenta showed acute villitis and intervillositis, the membranes showed evidence of chorioamnionitis, and the umbilical cord showed evidence of funisitis (Figure 3). At routine follow-up, she was doing well with no complaints.

## Discussion

Listeriosis is a rare foodborne febrile illness that has a unique predilection for the pregnant population. Listeriosis during pregnancy can cross the placenta, resulting in vertical transmission to the fetus, which frequently results in adverse pregnancy outcomes. All three patients reported in this series had no medical comorbidities or pregnancy risk factors. Most cases of listerial infections occur in healthy pregnant women [13]. In a review of more than 200 pregnant patients with listeriosis, fever was the most common symptom, followed by a flulike illness, and the third most common reported symptom was abdominal or back pain [4]. All three patients in this case series presented with fever and abdominal and/or back pain, and significantly, none of the patients reported a history of gastrointestinal tract symptoms. All patients had a high white cell count, a high absolute neutrophil count, and an absolute monocyte count. In the largest pregnancy-related listeriosis case review [4], the mean white cell count was 16,300 cells/uL.

A diagnosis of listeriosis requires a positive culture from the blood, cerebrospinal fluid, or placental fluid [14]. Listeriosis is not typically known to present with hepatitis. However, there are a few cases in the literature with reported listeriosis associated with hepatitis [15,16]. A histopathological examination of the placenta may reveal scattered, tiny yellowish lesions consisting of villous microabscesses with foci of necrosis and palisaded histiocytes. It is possible to see the Gram-positive bacilli in the focal villitis with neutrophils between trophoblasts and stromas [17]. Two of our patients had positive blood cultures confirming the *Listeria monocytogenes* organism, but because there was no blood culture collected for patient 2, the diagnosis was based on the clinical presentation, high serum leucocyte and monocyte counts, and the typical findings in placental histopathology. The other two cases showed no significant histopathological report. 

Several studies have reported that fetal listeriosis has a high mortality rate of 25%-35% [8,18]. In this case series, all cases resulted in fetal loss with 100% fetal mortality. Two patients already had an intrauterine fetal loss on presentation, confirmed by no fetal cardiac activity on ultrasound, and one of the patients had a viable fetus on presentation. However, she presented with a previable rupture of membranes secondary to the listerial infection. Two cases were in the second trimester and one in the third trimester. In the case series reviewing more than 200 pregnant patients, the most common means of diagnosis was blood or placenta culture [4]. In this series report, two cases were diagnosed using blood culture and placental histopathology, and one was diagnosed only by placental histopathology.

Because listeriosis is rare, there are no randomized trials that assess the optimal type and duration of antibiotic treatment. Most available data are based on reports of clinical experience [19]. Penicillin, ampicillin, and amoxicillin have been used extensively in the treatment of listeriosis, and most experts recommend 6 g or more per day of ampicillin for treatment in pregnancy [19]. In patients who are allergic to penicillin, trimethoprim/sulfamethoxazole offers an alternative treatment [19]. All patients in this series received intravenous (IV) ampicillin. Two of them were discharged on oral Augmentin (amoxicillin), and the third was discharged on trimethoprim/sulfamethoxazole.

Maternal outcomes of listeriosis are typically good; in the MONALISA study, which was a large prospective cohort study of clinical features and prognostic factors of listeriosis in France involving more than 800 confirmed listeriosis cases and their outcomes, 107 of the patients were pregnant, and there were no deaths among the pregnant patients [8]. However, fetal outcomes are much more serious, involving abortion, preterm birth, septicemia, CNS infection, or death [20]. In this case series, there was no maternal mortality or long-term morbidity in any of the patients; they were all discharged in good health and were doing well on follow-up.

## Conclusions

In conclusion, although the maternal outcome is often good, there is a high fetal and neonatal mortality rate associated with listeriosis during pregnancy. This is clearly reflected in the above cases of listeriosis in pregnancy, which highlight the severe consequences the infection can have on the fetuses, including miscarriage and IUFD. All pregnant women should be advised to avoid foods associated with *Listeria* infection as outlined in the CDC to prevent listeriosis in pregnancy; this includes unpasteurized dairy products, deli meats, and hot dogs. In view of the severe consequences that listeriosis infection can have during pregnancy, public health education and awareness campaigns have a crucial role in educating pregnant women about the importance of preventative measures including safe food during pregnancy and the necessary precautions to reduce the risk of infection.
